# How Does Health-Related Advertising with a Regulatory Focus and Goal Framing Affect Attitudes toward Ads and Healthy Behavior Intentions?

**DOI:** 10.3390/ijerph14121507

**Published:** 2017-12-04

**Authors:** Chia-Yen Lin, Wei-Ju Yeh

**Affiliations:** Department of Public Administration and Management, National University of Tainan, 33, Section 2, Shu-Lin St., Tainan 700, Taiwan; erickim1109@gmail.com

**Keywords:** regulatory focus theory, message framing, healthy advertisement, attitude toward healthy advertisement, behavior intention

## Abstract

The health costs of colorectal cancer have increased over the years in Taiwan. The National Health Insurance Administration (NHI) and the Health Promotion Administration of the Ministry of Health and Welfare (MOHW) in Taiwan advocate that people have to change their unhealthy behaviors; however, the number of patients of colorectal cancer is increasing annually. This research discussed the effects of healthy diet advocacy advertisements (ads) on healthy diet behavior intentions as influenced by the interactions between regulatory focus theory (RFT) and message framing effects. Both regulatory focus theory and message framing effect were discussed for the relationship between advertisement and behavior change in many fields, such as health-related behavior, pro-environmental behavior, consumer choice, etc. We executed an experiment with four different types of public health advocacy ads. A 2 (regulatory focus: promotion vs. prevention) × 2 (message framing: gain framing vs. loss framing) two-factor experiment was adopted, and 201 valid participants responded to the questionnaire. Results indicated that if the ad’s regulatory focus is promotion focus, viewers’ attitudes toward the ad and their behavior intentions are more positive when the slogan of the ad is gain framing rather than loss framing via the multiple analysis of variance (MANOVA), and vice versa. Respondents found the communication easier to comprehend when the ads evoked the respondents’ regulatory focus and applied the appropriate message framing, thus improving the efficacy of health-related advertising. We offer suggestions regarding the future use of health-related advertising for the MOHW.

## 1. Introduction

Unhealthy eating behavior and obesity are closely associated with cancer. In Western societies, cancer is thought to be related to the customary consumption of animal products, fats, and carbohydrates [1]. With the Westernization of eating behavior in Taiwan, the number of colorectal cancer patients has increased rapidly. According to the registry data in 2013 and 2014, 15,140 and 19,315 people were newly diagnosed with colorectal cancer, which are both the second highest among all cancers in Taiwan [2]. Colorectal cancer among men/women increased by an average of 21.5%/9.7% based on the analysis of the standardized colorectal cancer incidence rate from 2004 to 2013 [3]. For many years, the Health Promotion Administration, Ministry of Health and Welfare (MOHW) has advised people to change their dietary habits and to reduce their potential dietary risks (in addition to undergoing early screening tests). Several colorectal cancer prevention organizations—including the Taiwan Colorectal Cancer Alliance, Colorectal Cancer Care Network, and Formosa Cancer Foundation—also use advertisements to encourage people to maintain healthy eating behavior to prevent colorectal cancer. However, in previous colorectal cancer advertisements, the MOHW merely advocated screening tests and recommended three crucial colorectal cancer preventions (i.e., eat more whole grains, eat more fruits and vegetables, and exercise more frequently) or emphasized the negative consequences of colorectal cancer (e.g., informing people that cancer is a disease of modern civilization and that four colorectal cancer cases are diagnosed every 3 h) [4,5]. Despite the effort of those organizations, the age-standardized mortality rate of colorectal cancer was 14.9 per 100,000 people, which is the third highest fatal cancer in 2015 [3]. We therefore hypothesized that such advocacy was ineffective because the advertising content did not align with the viewers’ psychological states.

Previous studies of health-related messages or information communication have considered the framing effect as a crucial variable. The findings have been inconsistent; several studies have shown that positive framing delivers a more persuasive message [6,7], whereas numerous other studies have indicated that negative framing is more persuasive [8,9]. To explain these inconsistent findings, Lee and Aaker [10] applied regulatory focus theory (RFT) to message framing. RFT posits that people have two independent motivation orientations when pursuing goals: (a) promotion focus, which emphasizes the pursuit of goals or positive outcomes, and (b) prevention focus, which underscores the avoidance of losses or negative outcomes [11]. These two orientations correspond with the concepts of gain (e.g., positive/favorable) and loss (e.g., negative/unfavorable), respectively, in message framing. Some research on message framing [10,11] has suggested that promotion-and prevention-focused messages can be presented in either a positive or a negative frame, also known as a “gain versus loss” frame in some streams of literature [11]. Although previous studies have combined message framing with regulatory focus [12,13,14], few studies have directly included these two theories in a single advertisement. For instance, Zhao and Pechmann [15] attain social approval as a gain frame versus forgoing social disapproval as a loss frame with promoting a focused message of anti-smoking in their study. According to Mann et al. [13], most studies have adopted questionnaire surveys to determine participants’ focus orientations before asking them to watch an advertisement and evaluate its effectiveness. However, such an approach is difficult to adapt to real-life situations because marketers can only alter the cognition and behaviors of viewers through advertising appeals rather than through manipulative tactics. Therefore, the purpose of this study was to examine and verify the efficacy of manipulating viewers by asking them to read a health-related advertisements with regulatory focus and message framing.

### 1.1. Literature and Hypotheses

#### 1.1.1. Regulatory Focus Theory and Regulatory Fit

Higgins [16] proposed the regulatory focus theory (RFT), a self-regulatory system extended from self-discrepancy theory. RFT proposes that individuals compare their “actual self” with their “ideal self” or “ought self” and suggests that promotion focus occurs when one focuses on one’s ideal and that prevention focus occurs when one regards one’s goal as a duty or responsibility that should be fulfilled [17]. RFT has been applied to the field of consumer behavior in recent years. Bui and Krishen [18] examined the intersection of ideal weight goal progress and regulatory orientation on consumers’ health-related decisions, and they found the prevention-oriented individuals do not report the expected outcome of the goal persuasion.

Although most human beings want to accomplish beneficial outcomes by making the correct decisions, the regulatory orientations and strategies employed to achieve this goal differ. Regulatory fit theory maintains that people have different preferences for the strategies used to achieve their goals because of their different regulatory orientations. Promotion-focus-oriented people prefer to use eager strategies, which facilitate a positive outcome. Prevention-focus-oriented people prefer vigilant strategies, which facilitate a nonnegative outcome [19,20,21]. When the strategy adopted by an individual matches his or her regulatory orientation, a regulatory fit is achieved. Such a regulatory fit adds value to the actions that people execute, enhancing their motivation to achieve their goals [14,22,23] and promoting increasing positive emotions about their ideal choice and reinforcing the negative emotions about the non-ideal choices [24,25,26]. In addition, people experiencing a regulatory fit for themselves feel more affirmative about the decisions that they have made in the past [11,24,26] and attach a higher value to their goals [11,20,21].

#### 1.1.2. Message Framing

Message framing posits that people make different decisions according to how a single topic is presented [27]. Levin, Schneider, and Gaeth [28] identified three distinct types of message framing: risky choice framing, attribute framing, and goal framing. Risky choice framing indicates an individual’s willingness to assume risks. When messages are presented with positive framing, people tend to avoid risks by selecting the option exhibiting a certainty effect; conversely, in negative framing, people are inclined to select risky options because they are more willing to assume risks [29,30]. Attribute framing is generated when the attributes of an event are positively or negatively evaluated. In general, participants are more likely to respond positively to positive framing because the information therein is more favorable and generates positive associations; conversely, participants are less likely to favor negatively framed messages because they generate negative associations. Attribute framing is also frequently used to explore how consumer purchase attitudes and intentions differ according to product attributes [31]. Goal framing indicates the positive (negative) outcomes generated by engaging (not engaging) in a behavior; thus, the messages yield various degrees of persuasiveness. Goal framing and attribute framing differ in that goal framing focuses on the result of a behavior and both positive and negative framing achieve identical results, whereas attribute framing focuses on the attributes of the object itself. In general, messages generated through negative framing are more persuasive than those generated through positive framing. Message framing effect has been an interesting and significant research field in health message communication. Many studies have investigated how the effect of the message framing affects the persuasion of health message propaganda, and verified that the manipulation of the framing will lead to the change of healthy behavioral intention or behavior [32], for example, behavior intention to quit smoking [33]; screening on self-related health [34].

Message framing indicates that a message is presented either through positive or negative methods. Individuals’ assessments of the messages therein differ according to the manner in which the messages are framed, resulting in changes in individuals’ attitudes or behaviors. Many previous studies have explored whether advertisements should adopt gain or loss frames, and some of these studies have indicated that gain framing is more persuasive; other studies have indicated that loss framing is more persuasive. Thus, previous studies have reported inconsistent results [32]. In addition to message framing, the situation in which an individual is placed affects the level of persuasiveness.

#### 1.1.3. Interaction between Regulatory Focus Theory and Message Framing

RFT has been applied to further investigate which form of message framing enhances the persuasiveness of advertisements targeting viewers with distinct psychological traits. Previous studies have combined RFT with message framing [16,35,36]. Rothman and Slovey [37] indicated that message framing can be manipulated with RFT by using two methods. The first method involves determining whether advantages can be gained, which involves a gain-framing concept that indicates the advantages to be gained if a goal is accomplished and a loss-framing concept that indicates the advantages that cannot be gained if the goal is not accomplished. The second method involves determining whether negative outcomes can be avoided. Gain framing refers to the negative outcomes that are avoided, and loss framing refers to the negative outcomes that are obtained. The concepts of gain and loss in message framing are consistent with promotion focus and prevention focus in RFT; therefore, we infer that regulatory fit is generated and that it exerts a strong persuasive effect when gain framing is delivered to participants with a temporary promotion focus, whereas loss framing is delivered to participants with a temporary prevention focus.

**Figure 1:**
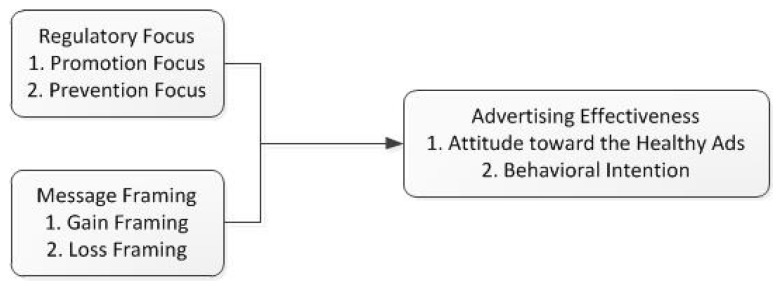
Research Framework.

The concept of regulatory focus has been verified by Lee and Aaker [10] in their health-related study involving a grape juice advertisement with various message frames and regulatory focuses. The results confirmed that when the advertisement manipulated the participants into a promotion focus orientation by emphasizing the high vitamin C and iron content of the grape juice, gain framing (i.e., to improving nutrition) for the advertising slogan was more effective than loss framing (i.e., not missing the opportunity to improving nutrition). Conversely, loss framing (i.e., not missing the opportunity to prevent cardiovascular disease) for the advertising slogan was more effective than gain framing (i.e., to prevent cardiovascular disease) for the slogan when the advertisement induced a prevention focus orientation by emphasizing the antioxidant content in the juice and its effect on cardiovascular disease prevention. The findings indicated that the advertisement induced fluent thinking and generated positive feelings when the message was oriented toward a regulatory fit; therefore, a more persuasive effect was generated. In addition, rather than exploiting the viewers’ regulatory focus orientations, the persuasiveness of the advertisement can be enhanced by steering viewers toward a temporary regulatory focus orientation and then delivering a framed message that fits their perceptions. Zhao and Pechmann [15] reported similar results in their study on antismoking advertisements targeting adolescents. The study suggested that social marketers could manipulate regulatory focus by their choice of the message context because we might not know anyone’s psychological orientation. Thus, a gain-framed message could be aired during a show that primes a promotion orientation and a loss-framed message could be aired during a show that primes a prevention orientation. The aforementioned studies show that persuasiveness can be enhanced when the presented message fits the regulatory focus of the intended audience [36]. Therefore, this study proposed the following hypotheses and research framework (Figure 1).

**Hypothesis 1a** **(H1a).** As content of an ad’s regulatory focus is promotion, viewers’ attitudes toward the ad are more positive when the slogan of the ad is gain framing rather than loss framing.

**Hypothesis 1b** **(H1b).** As an ad’s regulatory focus is prevention, viewers’ attitudes toward the ad are more positive when the slogan of the ad is loss framing rather than gain framing.

**Hypothesis 2a** **(H2a).** As content of an ad’s regulatory focus is promotion, viewers’ behavior intention is more positive when the slogan of the ad is gain framing rather than loss framing.

**Hypothesis 2b** **(H2b).** As an ad’s regulatory focus is prevention, viewers’ behavior intentions are more positive when the slogan of the ad is loss framing rather than gain framing.

Because of the hypothesis, we hereby discussed the “attitude towards healthy advertisement” and “healthy behavior intention” more detail in the next sub-section.

#### 1.1.4. Attitude towards Healthy Advertisement and Healthy Behavior Intention

Attitude is an important psychology concept that is widely applied for sociology and consumer behavior. People used the term ‘attitude’ in many contexts. An attitude is a lasting, general evaluation of emotion or actions toward people (including oneself), objects, advertisements, or issues [38]. In our study, we adopted the dimensions of perception and emotion to measure one’s attitudes toward the healthy ads. Some studies have discussed the positive relationship between attitude toward ads and the intentions. Yet, the relationship between attitudes and behavioral intentions often seems to be a controversial issue which is missing or weak in many research areas [39]. Recent advances of behavioral intention models, an extension of TPB (theory of planned behavior) and TRA (theory of reasoned action), offer new perspectives and possible explanations for the attitude–behavior gap, which are posited to have an influence on attitude formation, behavior intentions, and decisions making [40]. Some studies have focused on how to change people’s attitudes to improve their behavior intentions in health-related research topics, included ads that promote cessation of smoking in adolescents [41,42,43], eating the healthy food [44], and developing healthy exercise habits [45]. These studies in health-related fields have confirmed that attitudes and behavioral intentions are highly correlated, and also implicated when individuals with positive attitudes would commit themselves to a certain behavior are more likely to engage the certain behavior.

## 2. Materials and Methods 

### 2.1. Research Framework

This study adopted regulatory focus (i.e., prevention and promotion focus) and message framing (i.e., gain and loss framing) as the independent variables, whereas attitude towards the healthy ads (AttAds) and behavior intention (BI) were adopted as dependent variables to explore the advertising effects of message framing under distinct regulatory focus conditions. Figure 1 shows the research framework.

### 2.2. Participants

A total of 225 students from three universities consented to participate in the study and were randomly assigned to the four scenarios in this study. The majority of the students were female (58.2%); 77% of the participants were undergraduate students, and 22.4% were graduate students. The participants’ mean age was 24.4 years (range 16–56), and 70.1% of participants was under 25 years old. Regarding the fruit and vegetable eating behavior, the majority reported eating less than one cup of fruits and vegetables per day (52.5%), and an additional 32.2% of the participants ate less than two cups of fruits and vegetables per day. The eating behaviors in this study reflected the general unhealthy eating behaviors in Taiwan.

### 2.3. Measurements

We adopted the manipulation method used by Lee and Aaker [10] in their grape juice advertisement, and we primed the participants into temporary promotion or prevention focus orientations according to the content of the advertisements as the first step of the experimental design. In this study, the advantages of eating fruits and vegetables advocated by the Formosa Cancer Foundation and MOHW were used as a reference; specifically, the advocated message “can promote health” was adopted to evoke promotion focus, whereas the message “can prevent disease” was adopted to induce prevention focus. Then, as the second step of the experiment, we used gain framing and loss framing for the advertisement slogans involving promotion and prevention focus, respectively, based on previous research by Lee and Aaker [10]. We used the gain message “preventing colorectal cancer” and the loss message “missing out on preventing colorectal cancer” to be the slogans of an ad with a prevention focus to design the advertisement. We also manipulated the gain message in the context as “for a healthy and long life, you need to eat more fruits and vegetables” and the loss message as “you cannot live a long and healthy life if you do not eat more fruits and vegetables” when the advertising slogan was focused on promotion. In contrast, we manipulated the gain frame message as “you can avoid colorectal cancer by eating more fruits and vegetables” and the loss message as “you are more likely to get colorectal cancer by not eating fruits and vegetables” when the advertising slogan was focused on prevention.

The questionnaire on attitudes toward healthy advertisements (AttAds) was developed by Maheswaran and Meyers-Levy [46], with 11 questions designed to measure the participants’ cognition and affection toward the developed advertisements. Each question was measured using a seven-point Likert scale. Behavior intention (BI) refers to the probability of a person adopting a certain behavior [47], and numerous studies have employed BI to examine the effect of an ad or a message [41,42,44,45]. Therefore, we assessed the advertisement effects by measuring behavior intention and adopted the questionnaire design proposed by Updegraff et al. [48]; specifically, six questions were used to measure the participants’ behavior intentions after they read the advertisements in this study. Each question was measured using a seven-point Likert scale, in which higher scores indicated that stronger behavior intentions were generated after watching the advertisement.

### 2.4. Design and Procedure

This study adopted a 2 × 2 factor experimental design, in which promotion and prevention focus (i.e., regulatory focus) were combined with gain and loss framing (i.e., message framing); therefore, four types of color-printed advertisement were designed in this study. Participants were randomly assigned to one of the four scenarios, and the advertisements were identical in graphic and textual composition but differed in terms of content. Before the experiment commenced, the participants were informed that the advertisement was about improving healthy eating behavior and were also required to read the printed advertisement in detail. We provided a traditional paper questionnaire immediately after the participants viewed the advertisement. Then they answered the questionnaire designed to evaluate the attitude toward the healthy advertisement and the behavior intention after reading the advertisement. A total of 225 questionnaires were administered in September and October 2014, yielding 201 valid responses and 24 invalid responses. The questionnaires were classified as invalid when the respondent gave the wrong answers for the manipulation checks.

## 3. Results

### 3.1. Manipulation Checks

The following items measured whether the message framing manipulations were successful: “According to the title of the advertisement, which of the following content type best reflects the advertisement’s message?” (Promotion-focused content: “eat more fruits and vegetables to live a long and healthy life” vs. “not eating fruits and vegetables means not living a long and healthy life”; prevention-focused content: “eat more fruits and vegetables to avoid colorectal cancer” vs. “not eating fruits and vegetables means that you could easily get colorectal cancer”). Only the questionnaires with properly completed responses were classified as valid questionnaires.

We used another four questions to evaluate whether the regulatory focus manipulations were successful. Two questions involved the degree to which the participants agreed with the content that did not appear in the advertisements, whereas the other two questions involved the degree to which the participants agreed with the prevention- or promotion-focused content. Each question was measured by the seven-level Likert item scale including “extreme strongly disagree = 1, strongly disagree = 2, disagree = 3, neither agree nor disagree = 4, agree = 5, strongly agree = 6, and extreme agree = 7”. We analyzed the questionnaires with paired sample t to confirm that if the regulatory focus has been manipulated successfully. The results showed that the manipulations of the experimental design produced significant differences between promotion focus and prevention focus (*M_promotion_* = 5.53 > *M_prevention_* = 3.72, *t*(100) = 9.755, *p* < 0.001). Considering the prevention focus, the outcomes showed that manipulating the prevention focus obtained significantly statistical different results than manipulating the promotion focus (*M_promotion_* = 5.84 < *M_prevention_* = 3.39, *t*(99) = 11.849, *p* < 0.001). To verify the internal consistency, a reliability analysis was conducted and got good Cronbach α values for the attitudes toward the ad and behavioral intentions, which were 0.914 and 0.854, respectively. These values were both higher than 0.7, revealing that the questionnaire had a good level of internal consistency for the scale.

### 3.2. Hypothesis Testing

Table 1 and Table 2 shows the results of the two-factor multivariate analysis. Our study shows that the main effect of regulatory focus achieved marginal significance, with a *p*-value of 0.071 [49]. However, the main effect of message framing was not statistically significant or even marginally significant. In contrast, there was a significant interaction effect of regulatory focus and message framing on AttAds and BI (*F* = 9.547, *p* < 0.001).

**Figure 2:**
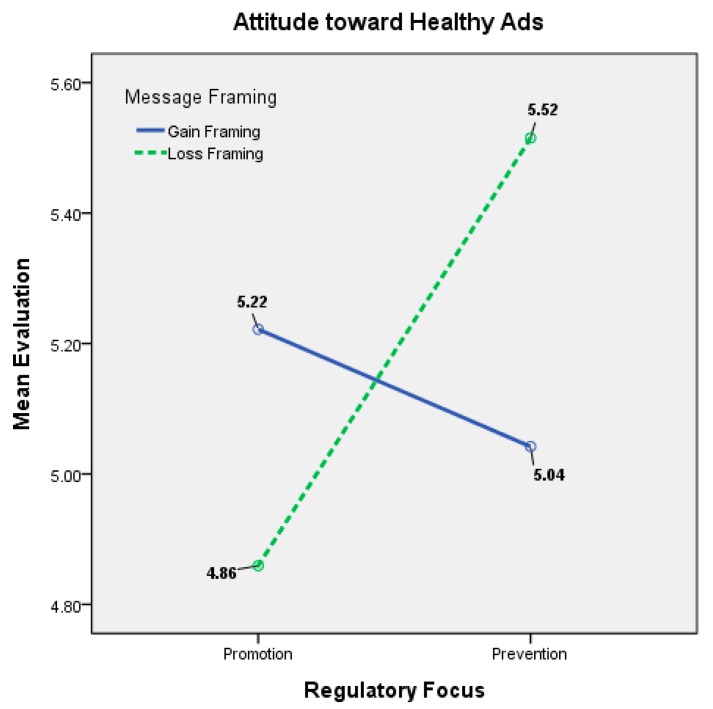
Interaction effects on attitude toward healthy ads between regulatory focus and message framing.

Regarding the interaction effects of regulatory focus and message framing on attitude toward healthy advertisements, the between-subject effect was significant. Then we conducted the independent sample *t* test to examine the means of two groups. The *t* test is a widely used market research statistics to test for differences between two groups of respondents, and the significance only when the *t*-value is bigger than the critical value of 1.708. When the temporary regulatory focus of the advertisement content was manipulated as promotion, the attitude toward the healthy advertisement containing a gain-framed message was superior to that of the loss-framed message (*M* = 5.2219 vs. 4.8593, *t*(99) = 2.273, *p* < 0.05), and the H1a was supported. When prevention was manipulated as the temporary regulatory focus, the loss-framed AttAds was superior to the gain-framed AttAds (*M* = 5.042 vs. 5.5152, *t*(98) = −3.616, *p* < 0.001), and H1b was supported. The statistically significant interaction effects indicated that H1a and H1b were supported (Table 3 and Table 4 and Figure 2).

**Figure 3:**
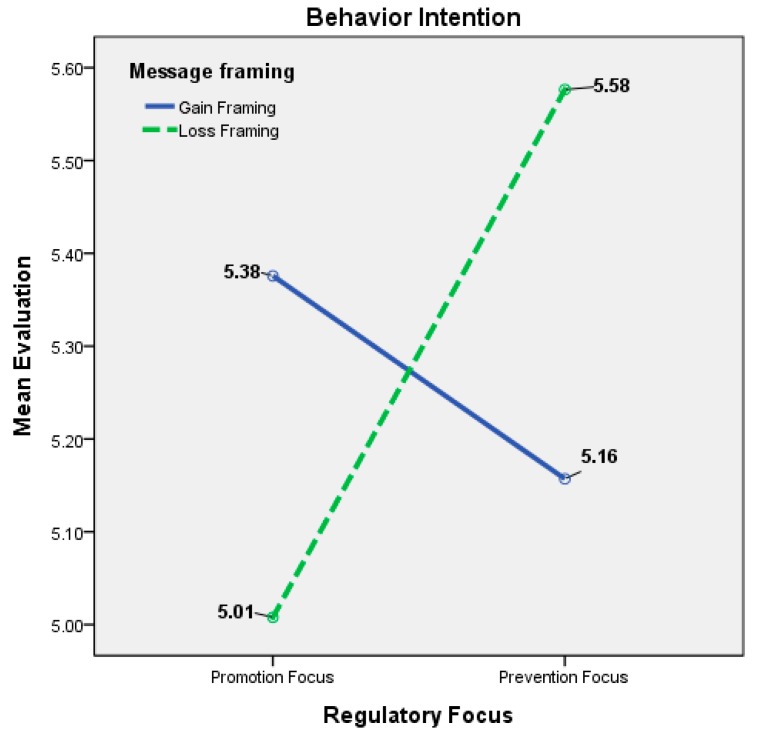
Interaction effects on behavior intention between regulatory focus and message framing.

Regarding the participants’ behavior intentions (Table 5 and Figure 3), when promotion was manipulated as the regulatory focus, the BI toward adopting the gain frame was superior to the BI toward adopting the loss frame BI (*M* = 5.3757 vs. 5.0079, *t*(99) = 2.480, *p* < 0.05). The difference was significant, and H2a was supported. When the regulatory focus was prevention, the BI toward using the loss frame was superior to the BI toward adopting the gain frame (*M* = 5.1571 vs. 5.5764, *t*(98) = −3.258, *p* < 0.001), and H2b was also significantly supported (Table 6).

## 4. Discussion and Policy Implication

The objective of consumer behavior research is to assist consumers in taking actions that are more rational; thus, if consumer behavior can be elucidated, the results can help policy makers and relevant organizations to develop effective advertising methods that encourage people to adjust their dietary habits. According to the Centers for Disease Control and Prevention (CDC) in the United States, unhealthy dietary habits have become the leading cause of death in the United States. The CDC has declared obesity an epidemic, unhealthy diet eating behavior is the second-leading actual cause of death [50]. To change unhealthy eating behavior intentions, the United States government has collaborated with private firms to advocate healthy dietary habits through marketing programs. For example, the government collaborated with fast food businesses to hold adolescent healthy lifestyle competitions. In response, these businesses have emphasized the benefits of eating healthy foods, such as salads, in their advertisements, which effectively increased the sale of salads. Therefore, adopting marketing strategies is an effective method to enhance health [50].

Our study is well structured and designed; the result is also very significant to public health. In this study, regulatory focus and message framing were combined and used to determine whether the manipulation of healthy advertisements can improve individual eating behavior intention. The purpose of this study was to determine whether the fit of message framing and regulatory focus in a healthy message advertisement could affect individuals’ attitudes toward healthy behaviors and change their eating behaviors intentions.

A regulatory focus effect and interaction effects of regulatory focus and message framing on the participants’ attitudes toward the healthy advertisement were observed, although the main effect of message framing was not statistically significant. However, the findings of this study supported all our hypotheses. The results verified that manipulating viewers or predicting viewer focus was unnecessary and that that the effectiveness of advertisements can be improved when the message framing fits the regulatory focus of the audience [16,37,51]. Thus, we provide empirical evidence to support the hypothesis that manipulating viewers through direct advertisement is feasible for health-advocating messages without the need to prime viewers to adopt a specific focus or to predict their focus using a scale test, which was consistent with the method proposed by Lee and Aaker [10].

In Taiwan, the incidence of colorectal cancer is increasing; however, most MOHW advertisements concerning colorectal cancer prevention directly appeal to the importance of the following two statements: (1) positive outcomes can be achieved through regular cancer screening tests, eating more fruits and vegetables, and exercising; and (2) negative outcomes can be generated if these three steps are not followed. However, the contents of these statements do not match the mental state of the audience. Currently, the MOHW considers these two statements to be critical methods for assisting people in preventing colorectal cancer, which indicates that regulatory-focused messages correspond with these key points. Based on the finding that regulatory focus was significantly correlated with message framing, we argue that future health-related advertisements should contain content that elicits various types of regulatory focus in viewers, and message framing and graphical and textual content should be adjusted according to the state of focus, which allows people to easily understand the message, thereby enhancing the overall advertisement effect. The government should produce healthy advertisements with a regulatory focus and message framing fit (i.e., promotion focus with gain framing messages or prevention focus with loss framing messages, which will improve the efficacy of health-related advertisements and change eating behavior intentions).

Public health advertisements are an important instrument for the government to persuade and change people’s healthy behavior intention. Our research provided strong evidence and suggestions for the government to make the public health advertisements more effective: the government has to integrate the promotion focus with gain message framing, or the prevention focus with loss message framing into advertisement content. We suggest that the interaction effect of prevention focus and loss message framing is the greatest of the four scenarios, the government may take this advertising strategy to enhance the people’s healthy behavior intentions to reach the goal of healthy nation.

## 5. Conclusions

Our research results differed slightly from those reported by Keller & Lehmann [52]. In their meta-analysis on health communication messages, the authors observed that the interaction effect between regulatory focus and message framing on behavior intention was superior only when promotion was the regulatory focus, whereas the behavior intention involving loss framing was not significant when prevention was the regulatory focus. Gallagher and Updegraff [53] conducted a meta-analysis on framed health messages using attitude, BI, and actual behavior as dependent variables. The authors reported that the effects of these three variables differed; specifically, the effect size of each variable was in the ascending order of actual behavior, attitude, and BI, which explains why the effect of attitude toward healthy advertisement was greater than that of BI in our current study.

To summarize, the current study confirmed that the viewers’ regulatory focus can be altered temporarily by the situation [10,16], and we could influence viewers’ attitudes and behavior intentions through advertisements with a temporary regulatory focus stimulation. The policy marketers should spend time to make salient or prime a single regulatory focus through their advertisement design rather than manipulate the viewers’ focus orientation. Our study also provides strong evidence that an alignment between a message’s regulatory focus and the message frame may facilitate message comprehension or accessibility and thus support the expectation in the achievement of healthy advertisement. We provide a significant result and strong support for future research that psychologists or scholars may not need to focus on matching implementation intentions with individuals’ regulatory orientations. The fit of temporary regulatory focus stimulation and message framing in a public service advertisement can also help health psychologists or government officials in deciding how to persuade people to develop better healthy eating intentions.

The current study has some limitations. First, we asked the participants to complete the questionnaire immediately after they had viewed the advertisements; therefore, we cannot measure whether the participants’ actual behaviors differed thereafter. In other words, in advertisements advocating disease prevention, whether actual behavior can be predicted by behavior during the experiment cannot be confirmed. Therefore, future studies are recommended to follow up on the actual behavioral changes of participants several months after viewing the advertisements. Second, we recommend including other dimensions related to health messages, such as risk perception. Previous studies have found that people perceiving high risks typically focus on negative outcomes, and they focus on positive outcomes when perceiving low risks [32,46,52]. The results correspond with regulatory focus because if a person feels threatened by an object or event, then a strategy in response to negative outcomes is generated; therefore, a loss-framed message prompts individuals to perceive a regulatory fit. Conversely, if people do not feel threatened, they tend to seek positive outcomes; therefore, a gain-framed message prompts people to perceive a regulatory fit.

## Figures and Tables

**Table 1:** Multivariate tests.

Effect	Value	*F*	Hypothesis df	Error df	Sig.
Regulatory focus	0.027	2.680	2	196	0.071 ^
Message framing	0.002	0.154	2	196	0.857
Regulatory focus × Message framing	0.097	9.547	2	196	0.000 ***

^ *p* < 0.1, *** *p* < 0.001.

**Table 2:** Tests of between-subjects effect.

Source	Dependent Variable	Type III Sum of Squares	df	Mean Square	*F*	Sig.
Corrected model	BI	8.631 ^a^	3	2.877	6.033	0.001 ***
	AttAD	10.784 ^b^	3	3.595	6.828	0.000 ***
Intercept	BI	5517.395	1	5517.395	11,570.454	0.000 ***
	AttAD	5270.040	1	5270.040	10,010.884	0.000 ***
Regulatory focus	BI	1.514	1	1.514	3.175	0.076 ^
	AttAD	2.802	1	2.802	5.323	0.022 *
Message framing	BI	0.033	1	0.033	0.069	0.793
	AttAD	0.151	1	0.151	0.288	0.592
Regulatory focus × Message framing	BI	7.665	1	7.665	16.075	0.000 ***
	AttAD	8.642	1	8.642	16.417	0.000 ***
Error	BI	93.940	197	0.477		
	AttAD	103.707	197	0.526		
Total	BI	5727.833	201			
	AttAD	5486.182	201			
Corrected total	BI	102.571	200			
	AttAD	114.491	200			

^a^ R Square = 0.084 (Adjusted R Square = 0.070); ^b^ R Square = 0.094 (Adjusted R Square = 0.080). ^ *p* < 0.1, * *p* < 0.05, *** *p* < 0.001.

**Table 3:** Tests examining between-subject effects on attitudes toward healthy ads.

Source	Measurement: Attitudes toward Healthy Ads				
	Type III Sum of Squares	df	Mean Squared	*F*	Sig.
Regulatory focus	2.802	1	2.802	5.323 *	0.022
Message framing	0.151	1	0.151	0.288	0.592
Regulatory focus × Message framing	8.642	1	8.642	16.417 ***	0.000
Error	103.707	197	103.707		
R-squared	0.094				

* *p* < 0.05, *** *p* < 0.001.

**Table 4:** Interactions on attitude toward healthy ads between regulatory focus and message framing.

Independent Variables	Regulatory Focus	Message Framing	Sample Size	Mean	S.D.	*t*-Value
Attitude toward ads	Promotion focus	Gain framing	59	5.2219	0.7863	2.273 *
		Loss Framing	42	4.8593	0.7956	
	Prevention focus	Gain framing	52	5.0420	0.7231	−3.616 ***
		Loss framing	48	5.5152	0.5692	

* *p* < 0.05, *** *p* < 0.001.

**Table 5:** Tests examining between-subject effects on behavior intention.

Source	Measure: Behavior Intention				
	Type III Sum of Squares	df	Mean Squared	*F*	Sig.
Regulatory focus	1.514	1	1.514	1.154 ^	0.076
Message framing	0.033	1	0.033	0.033	0.793
Regulatory focus × Message framing	7.665	1	7.665	16.075 ***	0.000
Error	93.940	197	93.940		
R-squared	0.084				

^ *p* < 0.1, *** *p* < 0.001.

**Table 6:** Interactions on behavior intention between regulatory focus and message framing.

Independent Variables	Regulatory Focus	Message Framing	Sample Size	Mean	S.D.	*t*-Value
Behavior intention	Promotion focus	Gain framing	59	5.3757	0.7169	2.480 *
		Loss framing	42	5.0079	0.7588	
	Prevention focus	Gain framing	52	5.1571	0.6863	−3.258 **
		Loss framing	48	5.5764	0.5925	

* *p* < 0.05, ** *p* < 0.01.
